# Validation and comparison of two prearrest prediction scores for in-hospital cardiac arrest survival

**DOI:** 10.1016/j.resplu.2026.101493

**Published:** 2026-09-14

**Authors:** Hritul Karim, Eva Piscator, Gabriel Riva, Karl Oskar Ekvall, Kasper G. Lauridsen, Therese Djärv

**Affiliations:** a Department of Anesthesiology and Intensive Care, Capio S:t Görans Hospital, S:t Göransplan 1, 112 19 Stockholm, Sweden; b Department of Medicine, Solna, Karolinska Institutet, Karolinska University Hospital, D1:04, 171 76 Stockholm, Sweden; c Centre for Resuscitation Science, Department of Clinical Science and Education KI/SÖS, Stockholm, Sweden; d Department of Medicine Solna, Karolinska Institutet, Stockholm, Sweden; e Department of Emergency Medicine, Capio S:t Görans Hospital, Stockholm, Sweden; f Department of Cardiology, S:t Görans Hospital, Stockholm, Sweden; g Research Centre for Emergency Medicine, Department of Clinical Medicine, Aarhus University Hospital, Aarhus, Denmark; h Department of Anaesthesiology and Critical Care Medicine, Children’s Hospital of Philadelphia, University of Pennsylvania Perelman School of Medicine, Philadelphia, USA; i Department of Acute and Reparative Medicine, Karolinska University Hospital, Stockholm, Sweden

**Keywords:** Prediction model, Risk score, DNR, DNAR, Withhold Life-Sustaining Treatment (WLST), Decision-aid, Decision tool

## Abstract

**Background:**

The optimal pre-arrest prediction model for survival with favorable neurological outcomes following in-hospital cardiac arrest (IHCA) to aid Do-Not-Attempt-Cardiopulmonary-Resuscitation-decisions remains uncertain. This study aimed to externally validate the Prediction of Outcome for In-Hospital Cardiac Arrest (PIHCA) score and the Good Outcome Following Attempted Resuscitation (GO-FAR) 2 score and compare their performance for IHCA survival prediction.

**Method:**

We collected data from IHCAs across three Swedish hospitals covering all care levels. We identified IHCAs through the Swedish Registry of Cardiopulmonary Resuscitation during 2015–2021 and extracted prediction model data through electronic medical records. Outcome measure was survival with favorable neurological survival defined as Cerebral Performance Category 1–2 at discharge, obtained from the registry. Prediction model performance was assessed with AUROC-curves (c-statistic), calibration coefficients and nonparametric calibration plots.

**Results:**

We identified 647 IHCAs of which 501 were eligible for inclusion. Overall, 28% had survival with favorable neurological outcome. AUROC for the PIHCA score was 0.73 (95% CI: 0.67–0.78), compared to 0.70 (95% CI: 0.65–0.75) for the GO-FAR 2 score. The 95% CI for difference of AUROC-score when PIHCA score was compared to GO-FAR 2 score was −0.01 to 0.06, i.e. not significant. The PIHCA score calibration coefficient was 0.69 (95% CI 0.51–0.88) compared to the GO FAR 2 score calibration coefficient of 1.02 (95% CI 0.73–1.34). The calibration plots were adequate for both scores, although with some degree of miscalibration at certain ranges for each model.

**Conclusion:**

PIHCA score and the GO-FAR 2 score performed well and comparatively for prediction of survival with favorable neurological outcome following IHCA in a Swedish setting. Both scores could be considered for clinical use, in addition to sound clinical judgment, where the PIHCA score seems more adapted for clinical use.

## Introduction

Despite increased survival following in-hospital cardiac arrests (IHCA) over recent years, most resuscitation attempts remain unsuccessful.^1^, ^2^ As physicians are expected to withhold futile or potentially harmful interventions that do not align with patients’ goals of care,^3^ Do-Not-Attempt-CardioPulmonary-Resuscitation (DNACPR)-orders have become part of routine in-hospital care.^4^ However, making informed DNACPR-decisions requires an accurate understanding of the patients’ likelihood of survival. Available evidence indicates that physicians’ predictions of survival following IHCA are unreliable and in some cases no better than chance.^5^, ^6^ Furthermore, in a survey study with brief summarized cases prearrest prediction among physicians showed a wide range for the same patients, with significant inter-rater variability in DNACPR-decisions.^7^ Prediction difficulties have been reported as a key impediment to discuss DNACPR-decisions with patients and relatives.^8^ Additionally, patients tend to significantly overestimate their chances of survival and are strongly influenced by communicated survival probabilities.^9^ Without evidence-based estimates of survival, clinicians risk both withholding resuscitation from patients who might survive and providing resuscitation to patients who are highly unlikely to benefit.

In a scoping review on DNACPR practices across the UK, major inconsistencies in DNACPR decision-making and implementation practices were identified, partly because of difficulties in prediction of outcomes.^10^ The authors concluded that future research should look into ‘the utility of prediction tools to reduce uncertainty and variation in decision-making’. Moreover, the importance of making appropriate DNACPR-decisions is emphasized by the International Liaison Committee On Resuscitation (ILCOR) ‘Ten Steps Toward Improving In-Hospital Cardiac Arrest Quality of Care and Outcomes’ as a part of setting patient-centered goals of care and appropriate prioritization of resources.^11^

Over time, several attempts have been made to develop prediction scores of survival after IHCA based on pre-arrest factors to serve as a decision-aid for DNACPR discussions.^12^ However, an ILCOR systematic review found that only two prediction models demonstrated adequate performance together with alignment with the widely accepted outcome of ‘survival to discharge with favorable neurological status (Cerebral Performance Category (CPC) 1–2)’.^13^, ^14^ These were the Good Outcome Following Attempted Resuscitation (GO-FAR) 2 score^15^ and the Prediction of Outcome for In-Hospital Cardiac Arrest (PIHCA) score.^16^ Since then, the GO-FAR 2 score has undergone external validation in two cohorts,^17^, ^18^ whereas the PIHCA score has not yet been externally validated. Moreover, the predictive performance of these two scores has previously not been compared.

The objective of this study was to externally validate the GO-FAR 2 and PIHCA scores and compare their performance, with the aim of identifying a suitable tool for future clinical implementation.

## Material and methods

### Study design

This was an external validation and comparison of two prediction scores through a multicenter cohort study, reported in accordance with the TRIPOD-statement (Appendices C).^19^ The study was set to include a cohort of index IHCA in adult patients (≥18 years) from different settings, hospitals and time periods. The total cohort was compiled from three hospitals in Sweden, in two different regions: Capio Sankt Görans Hospital (2015–2020, local hospital, approx. 300 beds in Stockholm), Karolinska University Hospital (2017–2019, University hospital with two sites, approx. 1200 beds in Stockholm), and Skaraborgs Hospital (2019–2021, County hospital, approx. 400 beds, Western region of Sweden). The study was approved by Swedish Ethical Review authority through the regional ethics review board in Stockholm: 2013/1959-31/4 with amendments 2014/2064-32, 2015/971-32 and 2021-06596-02.

### The prediction scores

#### GO-FAR 2 score

The original GO-FAR score was developed in 2013 with data from Get-with-the-Guidelines-Registry compiling 13 variables predicting likelihood of survival with excellent neurological outcome defined as CPC 1 at discharge.^20^ The model was updated in 2020 to predict the more generally accepted outcome of survival with favorable neurological outcome defined as CPC 1–2 at discharge.^15^ It was simultaneously revised and named GO-FAR 2 score (Appendices, Table A),^15^ and has since then been externally validated.^17^, ^18^ The model categorizes survival with favorable neurological outcome as either very poor (0–5%), poor (6–10%), average (11–30%), or above average (>30%).^15^

#### PIHCA score

The external validation of the GO-FAR score in Sweden in 2018 showed miscalibration,^21^ and prompted the development of the PIHCA score, derived from an IHCA cohort in Stockholm county in 2013–2014 (Appendices B).^16^ The PIHCA score consists of 9 variables, and the sum of all variables predict the likelihood of favorable neurological outcome (CPC 1–2) at hospital discharge. Each predicted patient is assigned a percentage (0–100%) for likelihood of survival with CPC 1–2 at discharge.

#### Data collection

All cases were identified through the Swedish Register for Cardiopulmonary Resuscitation (SRCR), where the inclusion criteria was loss of consciousness with abnormal (or absent) breathing within the walls of a hospital prompting chest compressions and/or defibrillation.^22^, ^23^, ^24^ The SRCR is a validated national quality registry that reports IHCA from 96% of Swedish hospitals according to the Utstein style since 2005.^24^, ^25^ Completeness of case finding in SRCR is approximated to 85%.^26^ We excluded cases with an existing DNACPR-order, out-of-hospital cardiac arrests, IHCAs with ST Elevation Myocardial Infarction admitted directly to PCI from ambulance arrival, and lastly Emergency-Department IHCA in triage patients before physicians’ evaluation i.e. before admission. The main reason to exclude STEMI-patients and Emergency-Department IHCA prior to physician attendance was that they are not the intended target for the prediction scores.

#### Data extraction

Identified cases were linked using the personal identification number (unique identifier for all habitants in Sweden) to Electronic medical records which was manually reviewed for all variables for both prediction scores and collected in a standardized Microsoft Excel® spreadsheet. As reported by Kaji et al.,^27^ manual chart review is vulnerable to bias, and they concomitantly describe several strategies to mitigate potential pitfalls. Many of these vulnerabilities were addressed in this study. First, both prediction scores primarily comprise binary variables with clearly defined criteria, thereby reducing the risk of measurement error. Furthermore chart reviewers for each hospital were residents in internal medicine, cardiology or emergency medicine with more than three years of clinical training. All uncertainties during manual review prompted secondary review with study investigators. Additionally, the clinical outcome variable (CPC 1–2 at discharge or not) for both prediction scores i.e. was retrieved from the SRCR.

#### Data handling

No preprocessing was performed. Predictions were calculated according to the original validation model specifications for GO-FAR 2 and PIHCA score,^15^, ^16^ however without the intercept for the GO-FAR 2 score as it was not reported and requests to obtain it remain unresponded.

Two variables were modified compared to the original study on GO-FAR 2 score to avoid frequent missing values in accordance with definitions previously used,^21^ and one variable was relabeled in the PIHCA score:

(1) Hepatic insufficiency: As blood levels of Aspartate Transaminase (AST) and Alanine Transaminase (ALT) are not investigated on daily basis on all in-hospital patients in Sweden but rather on clinical suspicion or deterioration, they are frequently lacking and thus in this study assumed normal if missing, i.e. no evidence of hepatic insufficiency.
(2) Hypotension or hypoperfusion: the time frame for measurement of evidence thereof was expanded from <4 h to <12 h, to avoid frequent missing values.
(3) Neurologically intact at admission: As this variable is defined as Glasgow Coma Scale 15, we relabeled the variable in this study to “Alert and conscious at admission” to make a more clear distinction from similar variables assessing neurological function with CPC which reflect a more long-term neurological function.

There were no other differences in variable definition for GO FAR 2 score in this cohort compared to the original cohort. The comorbidities that compile the Charlson Co-morbidity Index (CCI) (Appendices D) were retrieved from the list of diagnoses (registered as ICD-10 codes) in the electronic medical records.

### Statistical analyses

As this was an observational study, a-priori sample size calculations were descriptive based on expected lengths of confidence intervals (CI) for the calibration slope, and the area under receiver operating characteristic (AUROC) curve. Appropriate sample sizes were determined using methods proposed by Riley et al. specialized to our setting.^28^ Assuming a skew-normal distribution for the linear predictors with parameters given by the maximum likelihood estimates using the development data for PIHCA score,^16^ suggested 500 cases for 95% CI widths of approximately 0.4 and 0.1 for calibration slope and AUROC curve, respectively; while 1000 cases gave expected CI widths of approximately 0.3 and 0.06, respectively (Appendices, Figs. A1 and A2).

Continuous variables were described by mean and standard deviation and compared between groups using bootstrap-based tests in simple linear regression. Binary variables were presented as counts and percentages and compared using chi-squared tests. Categorical variables were reported as counts and percentages and compared using likelihood ratio tests in logistic regression. Significance tests were two-sided with a significance level of 0.05. Analysis was performed using R version 4.5.2.

### Model performance

Model performance was assessed by AUROC-curve (c-statistic), comparison of AUROC using bootstrap-based confidence intervals, calibration coefficient, nonparametric calibration plots. The nonparametric calibration slopes give a detailed assessment of calibration throughout the model scale. For c-statistic, 0.5 indicates no discrimination whereas 1.0 indicates perfect discrimination. In general terms >0.9 indicates outstanding performance, >0.8 excellent performance, >0.7 good performance, >0.6 modest performance. However, each prediction model performance should be evaluated in its clinical context. We also tested PIHCA scores’ risk group categorization capacities for either “low predicted likelihood of neurologically intact survival” or “above-low predicted likelihood of neurologically intact survival” at different thresholds, where patients are categorized either below (low) or above (above low) the set thresholds of: 1%, 3%, 5%, 10%, 15%. Heterogeneity in calibration among hospitals was assessed by nonparametric calibration plots by hospital. Data were inspected for outliers to assess the possibility of erroneous records; none were identified.

### Missing data

Missing data were treated as missing at random for all except hepatic insufficiency, where missing was assumed as normal (see data handling). For all other variables, one complete case analysis was performed and one with multiple imputation for missing values. The effects of imputing missing values were assessed by comparing the results. Missing values were imputed using the R package mice. Missing outcome was excluded, not imputed.

## Results

In total, the cohort provided 647 index IHCAs out of which 501 were complete (Table 1). Overall, 28% survived with favorable neurological outcome (CPC1-2). The characteristics of the IHCA cohort are described further in detail in Table 2.

**Table 1:** Study cohort – inclusions and exclusions.

**Hospital (time period)**	**S:t Görans Hospital (2015–2020)**	**Karolinska University Hospital (2017–2019)**	**Skaraborgs Hospital (2019–2021)**	**Total cohort**
Cases identified in SRCR	236	353	208	797
Cases excluded, total	38	83	29	150
Not meeting inclusion criteria (not meeting definition, not Index Cardiac Arrest, missing complete personal identity number)	8	18	2	28
Excluded due to preexisting DNACPR-order	2	6	7	15
Exclusion, OHCA	6	13	4	23
Excluded STEMI-patient	5	27	10	42
Excluded Emergency-Department Cardiac Arrest before admission	14	12	6	32
Other	3	7	0	10
Cases included, total	198	270	179	647
Complete cases, total	103	235	163	501

SRCR = Swedish Registry of Cardiopulmonary Resuscitation. DNACPR = Do-Not-Attempt-Cardiopulmonary-Resuscitation. OHCA = Out-of-hospital Cardiac Arrest. STEMI = ST-elevation myocardial infarction.

**Table 2:** Table of demographics and IHCA characteristics from the Swedish Registry of Cardiopulmonary Resuscitation (SRCR) and Electronic medical records.

	Complete cases (*n* = 300)* No. (%)	Missing data (*n* = 166)* No. (%)	***p*-value**
Survival with CPC 1–2	83 (28)	37 (22)	0.25^a^
Age, mean (SD)	72 (13)	73 (13)	0.36^c^
Male sex	192 (64)	117 (70)	0.19^a^

**IHCA characteristics**			
*Initial rhythm*			0.79^b^
VF	40 (13)	5 (14)	
pulseless VT	33 (11)	5 (14)	
PEA	110 (37)	10 (29)	
Asystole	117 (39)	15 (43)	
***Location of IHCA***			
– General ward (8)	138 (46)	103 (62)	
– Coronary care unit (1)	46 (15)	9 (5)	
– Intensive care unit (2)	33 (11)	14 (8)	
– High-dependency unit/Intermediate care unit (7)	23 (8)	7 (4)	
– Emergency department (4)	21 (7)	12 (7)	
– Coronary catheterization lab (6)	18 (6)	6 (4)	
– Operating theater (3)	10 (3)	1 (1)	
– Outpatient clinic, radiology, lab (5)	8 (3)	7 (4)	
– Other (8888)	3 (19	7 (4)	

**Predictor variables**			
***PIHCA score***			
Alert and conscious at admission	59 (20)	31 (23)	0.46^a^
Medical non-cardiac admission	127 (42)	85 (52)	0.06^a^
Sepsis	92 (31)	43 (27)	0.48^a^
Pneumonia	44 (15)	31 (19)	0.28^a^
Hypotension	71 (24)	21 (14)	0.02^a^
Respiratory insufficiency	124 (41)	65 (43)	0.89^a^
Acute kidney injury	106 (35)	49 (31)	0.36^a^
CCI, mean (SD)	2.10 (2.10)	2.34 (2.30)	0.22^c^
***GO FAR 2 score***			
CPC > 1–2 at admission	40 (13)	24 (15)	0.75^a^
Surgical cardiac admission	22 (7)	3 (2)	0.02^a^
Hematological or metastatic malignancy	32 (11)	20 (12)	0.73^a^
Septicaemia	22 (7)	17 (10)	0.32^a^
Renal insufficiency	54 (18)	30 (20)	0.77^a^
Hepatic insufficiency	38 (13)	17 (10)	0.58^a^
Pneumonia	As above	As above	
Hypotension	As above	As above	
Respiratory insufficiency	As above	As above	

* Restricted to cases from S:t Görans Hospital 2015–2020 and Karolinska University Hospital 2017–2019). IHCA characteristics data from SRCR for Skaraborgs Hospital have not been provided. IHCA = In-Hospital Cardiac Arrest. CCI = Charlson Co-morbidity Index (Appendices D).

a Chi-squared test.

b Global LRT.

c Bootstrap.

### C-statistic and ROC curves

The c-statistic for the PIHCA score was 0.73 (95% CI 0.67–0.78) and 0.70 (95% CI 0.65–0.75) for the GO-FAR 2 score. When comparing the PHICA score to the GO-FAR 2 score, the 95% confidence interval for the difference in c-statistics was −0.01 to 0.06, indicating no statistically significant difference on the 5% level between the scores discrimination performance. See Fig. 1 for comparison of ROC-curves for PIHCA and GO-FAR 2 score.

**Fig. 1:**
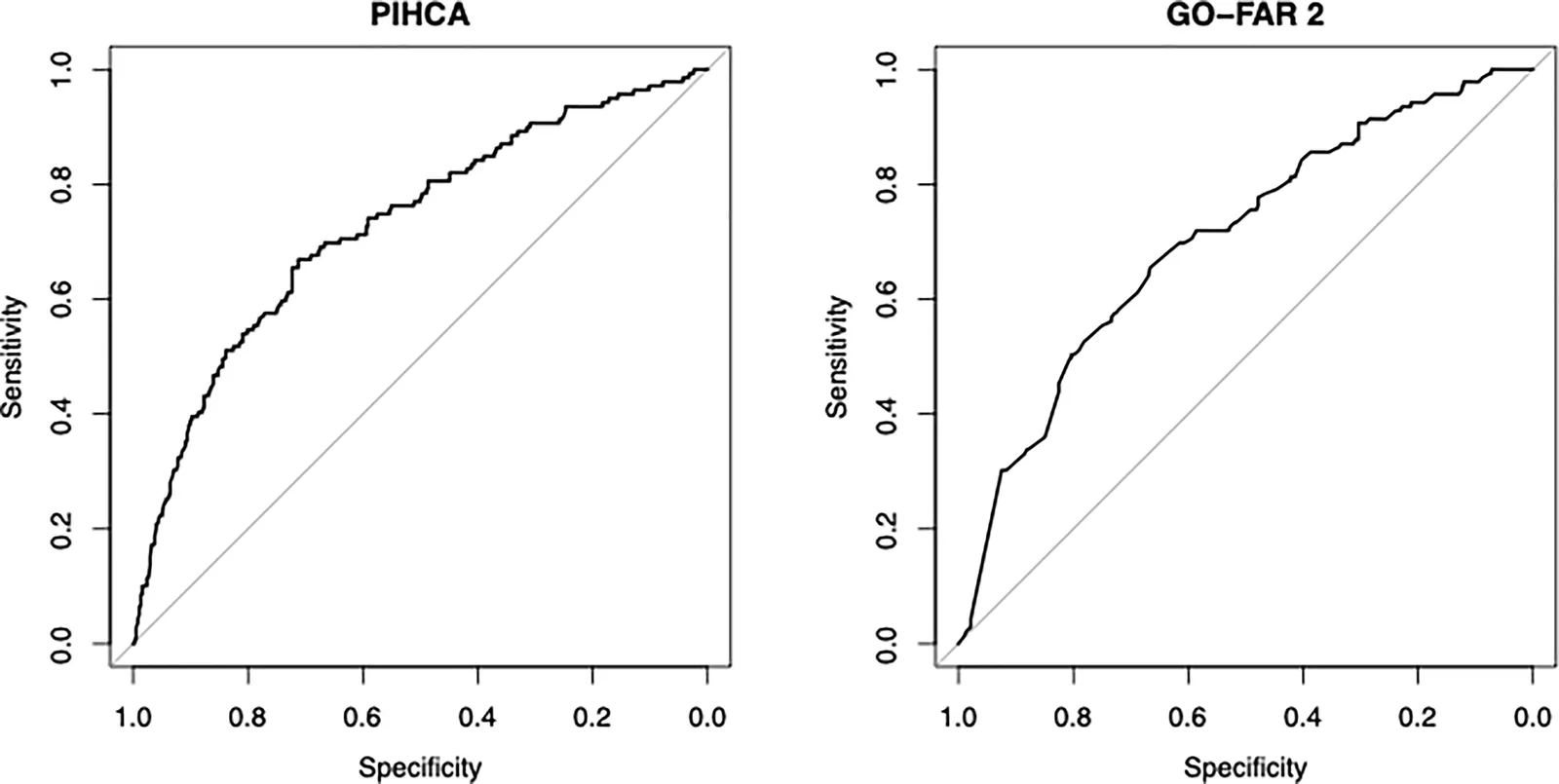
**AUROC-curve for PIHCA score and GO-FAR 2 score**.

### Calibration coefficients and nonparametric calibration slope

The estimated PIHCA score calibration coefficient was 0.69 (95% CI 0.51–0.88). The estimated GO-FAR 2 score calibration coefficient was 1.02 (95% CI 0.73–1.34).

The PIHCA score nonparametric calibration plot is shown in Fig. 2A. It shows an overall good calibration across the range compared to the reference line, except in lower predictions around 3–12%. In this range, observed survival outcomes (CPC1-2) are somewhat higher than predicted, and chances thus underpredicted by the PIHCA score, however with confidence interval including reference. The GO-FAR 2 score nonparametric calibration plot is shown in Fig. 2B. It shows a fair calibration across the range. In the range of 40–70% it demonstrates overestimation of outcome.

**Fig. 2A:**
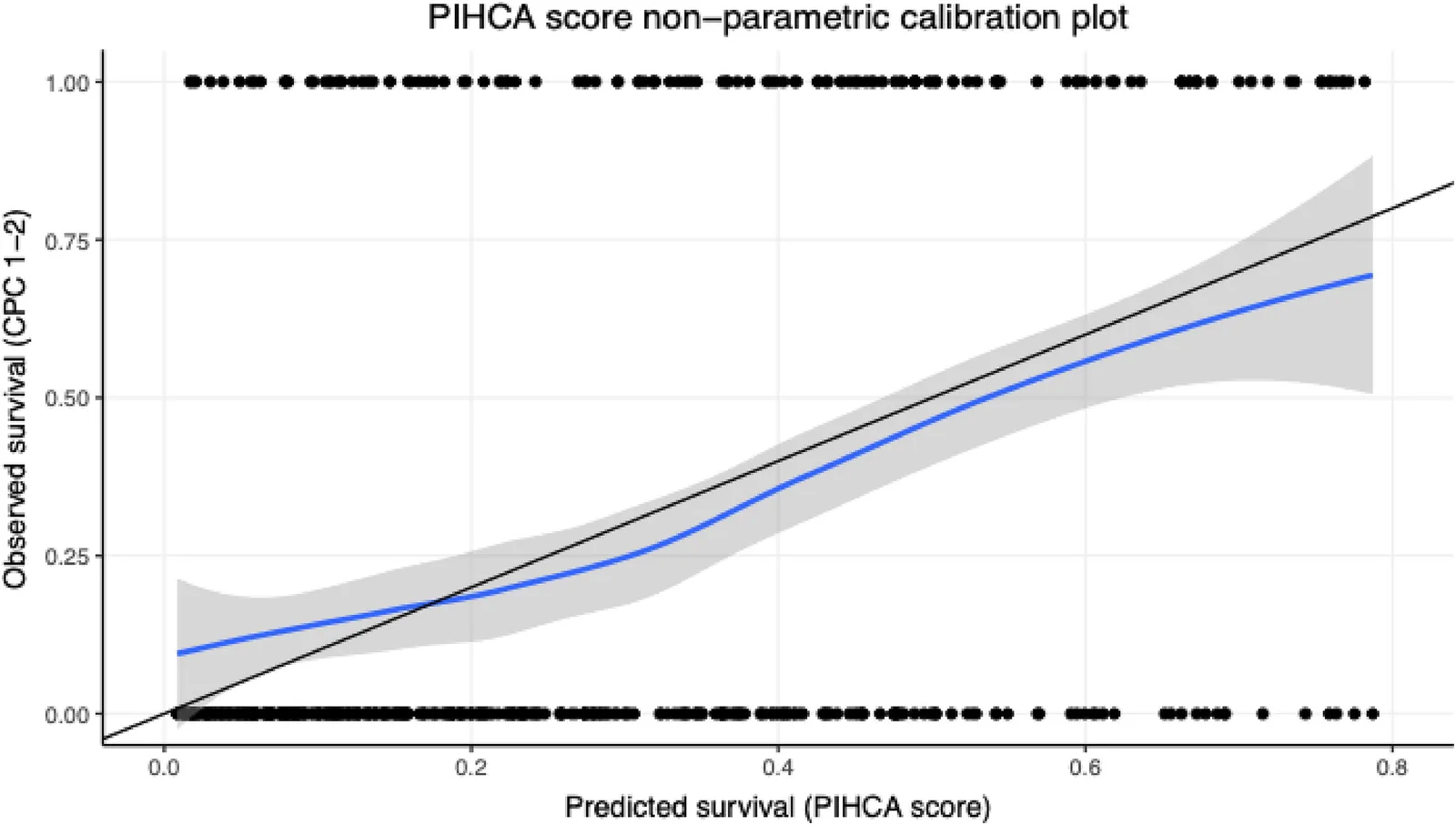
**PIHCA score nonparametric calibration plot**. CPC = Cerebral Performance Category.

**Fig. 2B:**
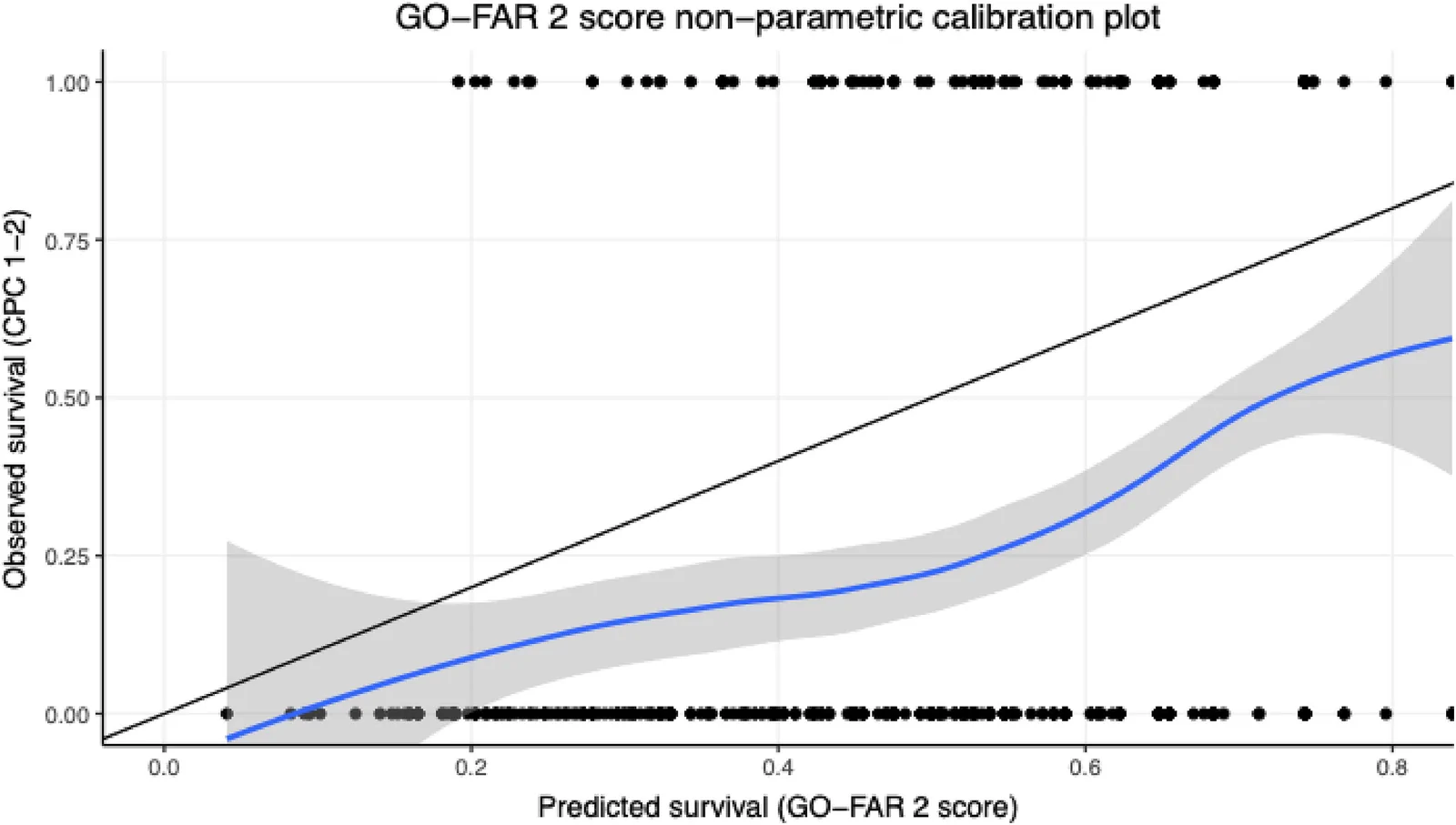
**GO-FAR 2 score nonparametric calibration plot**. CPC = Cerebral Performance Category.

### Results by hospital

Data heterogeneity between hospitals was assessed with nonparametric calibration plots for PIHCA score and GO-FAR 2 score by hospital (Appendices, Figs. B1 and B2^),^ although not formally tested. These indicated small to moderate differences, where both models generally overestimate survival at the Karolinska University Hospital. Sankt Görans Hospital and Skaraborg Hospital generally seem to be a better fit for this model, with smoother lines more parallell to the reference lines.

### Risk group categorization, negative and positive predictive values, sensitivity and specificity for PIHCA score

The Negative Predictive Value (NPV), Positive Predictive Value (PPV), Sensitivity and Specificity at these different thresholds for PIHCA score are displayed in Table 3. The NPV at 3% cut-off is 88%.

**Table 3:** Risk group categorization for PIHCA score.

**Threshold values**^a^	**Predicted outcome,** ***n* total = 647**	**True good outcome (CPC1-2),** ***n* total = 173**	**True poor outcomes (CPC > 2),** ***n* total = 474**	**NPV** **(95% CI)**	**PPV** **(95% CI)**	**Sens** **(95% CI)**	**Spec** **(95% CI)**
Low: <1%	2	0	2	100% (16–100)	27% (24–31)	100% (97–100)	0.002% (0,1–2)
Above-low: >1%	509	139	370				

Low: <3%	33	4	29	88% (72–97)	28% (24–33)	97% (93–99)	8% (5–11)
Above-low: >3%	478	135	343				

Low: <5%	57	6	51	90% (79–96)	29% (25–34)	96% (91–98)	14% (10–18)
Above-low: >5%	454	133	321				

Low: <10%	134	15	119	89% (82–94)	33% (28–38)	89% (83–94)	32% (27–37)
Above-low: >10%	377	124	253				

Low: <15%	195	27	168	86% (81–91)	35% (30–41)	81% (73–87)	45% (40–50)
Above-low: >15%	316	112	204				

CPC = Cerebral Performance Category.

NPV = Negative Predictive Value.

PPV = Positive Predictive Value.

Sens = Sensitivity.

Spec = Specificity.

a A threshold to categorize predicted outcomes as either low chances of favorable outcome, or above-low.

### Imputed data analysis

The number of missing values per hospital are shown in Table 2. Sensitivity analysis with imputed data were comparable with the main findings.

## Discussion

This study represents the first comparison of the two most promising pre-arrest prediction scores, as well as the first external validation of the PIHCA score. In this Swedish cohort, PIHCA score and GO-FAR 2 score performed well and comparatively, without statistically significant differences in performance metrics. Both models demonstrated some degree of miscalibration in certain ranges of each model.

In its first external validation, the PIHCA score demonstrated overall good performance. Calibration was adequate across most of the range, although some underestimation was observed in the first portion of the slope, which is a range where many patients are considered for DNACPR-decisions i.e. which could have clinical implications by underestimating survival in many patients. Compared with the original validation study which reported a c-statistic of 0.81,^16^ the PIHCA score performed somewhat worse in this cohort. Similarly, in the risk group categorization the NPV at 3% cut-off was 88%, compared to 97% previously. This implies that approximately one in eight patients categorized as non-survivors at this threshold nevertheless survived with favorable neurological outcome, an outcome which may be clinically unacceptable if cut-offs were to be used. Although the study populations were geographically similar, sensitivity analysis indicated that Karolinska University Hospital patients differ slightly from the other two hospitals. This is unexpected as the original PIHCA validation cohort included patients from the Karolinska University Hospital, however it could be explained by a major shift in hospital profile in October 2018 when Karolinska University Hospital reduced admissions,^11^ to include only selected patients with certain malignancies or advanced surgical requirements. This could contribute to differences in hospital cohorts where the drop in performance could in turn be explained by overfitting, arguably supported by the weak calibration coefficient. Moreover, the observed underestimation may also reflect improvements in survival over time compared to the development cohort,^29^ possibly attributable to general advances in in-hospital care, including enhanced surveillance and monitoring.^30^ Importantly, they could also be explained by increasing use of DNACPR-orders. These findings reinforce the importance of temporally updating prediction models, as determinants of IHCA outcomes evolve.

The GO-FAR 2 score here is both geographically and temporally validated and performed similarly to its original validation study reporting AUROC of 0.69 in its validation set,^15^ adding strength to previous conflicting results.^17^, ^18^ Notably, it demonstrated a better calibration coefficient than the PIHCA score which is based on a similar cohort. As mentioned, we believe this is likely explained by overfitting in the PIHCA score. Importantly however, it should be kept in mind that this summary statistic encompasses the whole scale whereas clinically only the lower half of the scale is of importance. The GO FAR 2 calibration plot shows some miscalibration, particularly in the higher predicted range, overestimating survival around 40–70%, although the plot is not fully interpretable without its intercept which we have tried to obtain. This underscores the need for local validation and recalibration of prediction models prior to clinical implementation.

In direct comparison, the PIHCA score and GO-FAR 2 score performed comparatively in this setting. However, from a clinical perspective, when predicting prearrest outcomes we would argue that only the first portion of a survival probability scale is clinically relevant as this is generally where DNACPR-decisions would be aided, wherefore stronger emphasis should be put in the nonparametric calibration plot as the calibration coefficient reflects a summarized measure for the whole scale of the model. Thus, with regards to performance metrics both could be considered for clinical implementation in this setting, however clinical usability aspects need to be taken into account. The PIHCA score is compiled mostly of variables readily available at the bedside when making decisions, however the CCI as a research tool could be a new element for clinicians however simply based on existing conditions. Whereas in the GO FAR 2 score, as reported in the previous external validation by Piscator et al.^21^ liver enzymes are to a large extent missing at the bedside in our setting as it is not evaluated daily but only on clinical suspicion of insufficiency. Furthermore, the CPC variable is from the Get-with-the-guidelines registry and mainly used in research and not in clinic, and assessments would be attempted by many infrequent assessors. For these reasons we would argue the PIHCA score to be more suitable for clinical introduction.

A validated pre-arrest prediction score has the potential to reduce variability and inconsistencies in DNACPR decision-making owing to uncertainty in survival estimations.^5^, ^7^, ^10^ However, the present findings where PIHCA score drops in c-statistic suggest that finding a universal prearrest prediction model that performs exceptionally well across all settings might be difficult to achieve, as even the most promising models have demonstrated mixed or modest performance in external validations.^17^, ^21^ This limitation may be due to the wide range of IHCA outcome determinants, many of which are not patient-specific (e.g. institutional DNACPR policies) or inherently unpredictable (e.g. initial rhythm, witnessed status). Nevertheless, despite these limitations, such models may still offer value as long they outperform clinician-based predictions and reduce physician-dependent variability in decision-making. Another consideration is that precise probability estimates may be less crucial than providing stable and reasonably accurate risk stratification.

From a clinical usability perspective, fixed decision thresholds for CPR (e.g. 1% or 3%) have previously been suggested.^13^, ^16^ However, the DNACPR-decision inherently depends on a balance between potential harms and benefits, which is uniquely contextualized for each patient. A given survival probability may be acceptable in one context but not in another, limiting the applicability of universal thresholds. Furthermore, such cut-offs require high negative predictive value to not misclassify potential survivors, while maintaining acceptable specificity. In the present analysis, no threshold achieved a satisfactory balance of these properties. We therefore argue against the use of fixed decision thresholds in this context.

Several general limitations of prediction models in this setting should be considered. As previously highlighted by Piscator et al.,^16^ selection bias arises in models derived from IHCA cohorts in systems where DNACPR-orders are routinely used. Patients most likely to have DNACPR-orders – presumably older, frailer and with greater comorbidity – are excluded in the analysis, which may lead to overestimation of survival after CPR. However, it is difficult to avoid this issue as DNACPR-orders are integral to routine clinical practice in many healthcare systems. Additionally, resuscitation efforts are often terminated based on perceived futility, meaning that observed outcomes reflect prevailing clinical judgements. Prediction models derived from such data risk reinforcing these judgements, potentially creating a self-fulfilling prophecy. One potential mitigation strategy would be to standardize the duration of resuscitation attempts and termination of resuscitation decisions, but as Swedish hospitals follow European Resuscitation Guidelines no such standardization or rules are systematically used in resuscitation practices. Rather, duration and termination are decided in each context by physicians in conjunction with the bedside team. Furthermore, as highlighted in the 2022 ILCOR systematic review,^13^ both prediction scores include physiological variables that may change over time, warranting model updates to reduce reliance on dynamic variables. However, the scores are intended to inform discussions with patients, families and colleagues rather than determine DNACPR-decisions in isolation. Prognostic estimates should be considered alongside clinical judgement, patient’s values and preferences, and the ethical and legal framework governing treatment limitation decisions. Consequently, minor changes in the score would not necessarily alter a DNACPR decision, whereas substantial changes reflecting a patient’s clinical course may appropriately prompt reassessment of treatment limitations. Altogether, clinicians should remain aware of the limitations inherent in these prediction models and avoid relying on them as a substitute for sound clinical judgement.

Despite these limitations, the current alternative with clinician-based predictions, are themselves subject to the same bias and lack precision as previously reported.^5^, ^7^ Prediction models may therefore offer a more objective and consistent framework for estimating survival and supporting DNACPR-discussions, provided they are not used in isolation. Ultimately, whether these prediction models can have a clinically meaningful impact on DNACPR-decisions remains a question for future implementation studies to address.

### Limitations

Generalisability of these results are likely limited to Scandinavian countries with comparable populations and health care settings. A larger geographical cohort including other Scandinavian hospitals could have provided more robust data, however from confidence bands and noted miscalibrations, it is not evident that additional cases would have impacted the results and conclusions. Retrospective manual review of electronic medical records is vulnerable to introducing bias in data, although this approach was necessary to capture variables not available in registry data.

## Conclusions

The PIHCA and GO-FAR 2 score performed comparatively in prediction of survival with favorable neurological outcome following IHCA in a Swedish setting. Regarding performance, both scores could be considered for clinical use, in addition to sound clinical judgment, where the PIHCA score seems more adapted for clinical implementation.

## Declaration of transparency/disclosure

Given their role as an Associate Editor, Kasper Lauridsen, had no involvement in the peer review of this article and had no access to information regarding its peer review. Full responsibility for the editorial process for this article was delegated to another journal editor.

## Research data

Datasets, analysis code can be made available upon request, with ethical permit.

## Declaration of usage of AI

During drafting of manuscript authors have used AI (ChatGPT) for text refinement and fluency. Content has thereafter been reviewed and edited, and authors take full responsibility for all contents.

## CRediT authorship contribution statement

**Hritul Karim:** Writing – review & editing, Writing – original draft, Software, Resources, Project administration, Methodology, Investigation, Funding acquisition, Formal analysis, Data curation, Conceptualization. **Eva Piscator:** Writing – review & editing, Methodology, Data curation, Conceptualization. **Gabriel Riva:** Writing – review & editing, Supervision, Project administration, Conceptualization. **Karl Oskar Ekvall:** Writing – review & editing, Writing – original draft, Software, Methodology, Formal analysis. **Kasper G. Lauridsen:** Writing – review & editing, Supervision, Resources, Project administration, Methodology, Investigation, Formal analysis, Conceptualization. **Therese Djärv:** Writing – review & editing, Supervision, Resources, Project administration, Methodology, Funding acquisition, Conceptualization.

## Funding

This work was supported by the Stockholm Region Council.

## Declaration of competing interest

The Prediction of Outcome for In-hospital Cardiac Arrest (PIHCA) score was developed by EP and TD. TD is editor at Resuscitation Plus, Editor-in-chief at Visual Journal of Emergency Medicine, Chair of Swedish Resuscitation Council, member of ERC ALS Committee, Chair of ILCOR First Aid Task Force, Leader of ILCOR Implementation of Ten Steps for IHCA. KGL is associate editor of Resuscitation Plus, and is vice chair of the ILCOR Education and Implementation Task Force.

## Appendix A Supplementary material

Supplementary Data 1 Tables, Figures and TRIPOD-Checklist.
