# Supplementary material for: Validation and comparison of two prearrest prediction scores for in-hospital cardiac arrest survival
Source: Resusc Plus. 2026 Sep 14;32:101493. doi: 10.1016/j.resplu.2026.101493 (PMC13634461; doi:10.1016/j.resplu.2026.101493)
Supplement: Supplementary file 1 [file mmc1.docx]

## Appendices

| **Variable** | **Definition** | **Comment** | **Source** |
| --- | --- | --- | --- |
| **Age** |  | Continuous | EMR |
| **Admission CPC <3** |  | Binary variable | EMR |
| **Septicemia** | Documented bloodstream infection in which antibiotic therapy has not yet been started or is still ongoing | Binary variable | EMR |
| **Medical non-cardiac admission** |  | Binary variable | EMR |
| **Surgical cardiac admission** |  | Binary variable | EMR |
| **Hepatic insufficiency** | Evidence of hepatic insufficiency within 24 h of the event, defined by total bilirubin >2 mg/dL (to convert to micromoles per liter, multiply by 17.104) and AST >2 times the upper limit of normal or cirrhosis | Binary variable | EMR |
| **Hypotension or hypoperfusion** | Evidence of hypotension within 4 h of the event, defined as any of the following: SBP <90 or MAP<60 mmHg; vasopressor or inotropic requirement after volume expansion (except for dopamine≤3µg/kg/min);or intraaortic balloon pump. | Binary variable | EMR |
| **Renal insufficiency or dialysis** | Requiring ongoing dialysis or extracorporeal filtration therapies, or serum creatinine >2 mg/dL (to convert to micromoles per liter, multiply by 88.4) within 24 h of the event | Binary variable. | EMR |
| **Respiratory insufficiency** | Evidence of acute or chronic respiratory insufficiency within 4 h of the event, defined as any of the following: Pao_2_/Fio_2_ ratio <300=(mmHgx0.1333kPa=<40), Pao_2_ <60 mmHg=8kPa, or Sao_2_<90% (without preexisting cyanotic heart disease); PaCO2, Etco2, or Tcco2 >50 mmHg=6.67 kPa; spontaneous respiratory rate >40/min or <5/min; requirement for NIV, or negative pressure ventilation (iron lung); or requirement for ventilation via invasive airway. | Binary variable | EMR |
| **Pneumonia** | Documented diagnosis of active pneumonia, in which antibiotic therapy has not yet been started or is still ongoing. | Binary variable | EMR |
| **Metastatic or hematologic cancer** | Any solid tissue malignancy with evidence of metastasis or any blood-borne malignancy | Binary variable | EMR |

**Table A.** Good Outcome Following Attempted Resuscitation (GO-FAR) 2 score (15).

EMR = Electronic Medical Records. CPC = Cerebral Performance Category. AST = Aspartate aminotransferase. SBP = Systolic blood pressure. NIV = Non-invasive ventilation.

| **Variable** | **Definition** | **Comment** | **Source** |
| --- | --- | --- | --- |
| **Alert and conscious at admission** | GCS 15.  If GCS not documented as such, information about the status of the patient as confused or disoriented in the electronic patient record will be interpreted as GCS<15. | Binary variable. | EMR |
| **Septicemia**  **Sepsis (53)** | Evidence of sepsis as a life-threatening organ dysfunction due to a dysregulated host response to infection, defined as at least 2 out of 3 of the clinical criteria qSOFA(ref 27 I Pisc): Altered mental status: GCS<15;  Hypotension: SBP≤100;  High respiratory rate: RR≥22/min (45) during the admission preceeding the CA. | Binary variable. | EMR |
| **Medical non-cardiac admission** |  |  | EMR |
| **Hypotension or hypoperfusion** | Evidence of hypotension within 12 h of the event, defined as any of the following: SBP <90 or MAP<60 mmHg; vasopressor or inotropic requirement after volume expansion (except for dopamine≤3µg/kg/min); or intraaortic balloon pump. | Binary variably. | EMR |
| **Renal insufficiency or dialysis** | Evidence of AKI defined as an absolute increase in serum creatinine by ≥26.5 µmol/L within 48h or an increase in serum creatinine to ≥1.5-fold baseline within previous 7 days. Baseline serum creatinine is defined as the median of creatinine values two-year preceding the CA (max 50 values). Cases without history of chronic kidney failure and unknown baseline kidney function should be assumed to have a baseline eGFR of 75 ml/min/1.73 m^2^ (according to CKD-EPI: eGFR = 141 * min(Scr/κ,1)α * max(Scr/κ, 1)-1.209 * 0.993Age * 1.018 [iffemale] * 1.159 [if black (46) . | Binary variable. | EMR |
| **Respiratory insufficiency** | Evidence of acute or chronic respiratory insufficiency within 12 h of the event, defined as any of the following: Pao_2_/Fio_2_ ratio <300=(mmHgx0.1333kPa=<40), Pao_2_ <60 mmHg=8kPa, or Sao_2_<90% (without preexisting cyanotic heart disease); PaCO2, Etco2, or Tcco2 >50 mmHg=6.67 kPa; spontaneous respiratory rate >40/min or <5/min; requirement for NIV, or negative pressure ventilation (iron lung); or requirement for ventilation via invasive airway. | Binary variable. | EMR |
| **Pneumonia** | Documented diagnosis of active pneumonia, in which antibiotic therapy has not yet been started or is still ongoing. | Binary variable. | EMR |
| **Age** |  | Continuous | EMR |
| **CCI (31, 32, 47-50)*** |  | Continuous | EMR |

**Table B.** Prediction of Outcome for IHCA (PICHA) score.
EMR = Electronic medical records. GCS = Glasgow Coma Scale. qSOFA = Quick Sequential Organ Failure Assessment. RR = Respiratory Rate. CA = Cardiac Arrest. SBP = Systolic Blood Pressure. MAP = Mean Arterial Pressure. AKI = Acute Kidney Injury. CKD = Chronic Kidney Disease. NIV = Non-Invasive Ventilation. CCI = Charlson Co-morbidity Index.

*see table D, appendices.

| **TRIPOD-Cluster Checklist** | **Section/row** |
| --- | --- |
| TITLE AND ABSTRACT |  |
| 1. Identify the study as developing and/or validating a multivariable prediction model, the target population, and the outcome to be predicted. | Title |
| 2. Provide a summary of research objectives, setting, participants, data source, sample size, predictors, outcome, statistical analysis, results, and conclusions. | Abstract |
| INTRODUCTION |  |
| 3a. Explain the medical context (including whether diagnostic or prognostic) and rationale for developing or validating the prediction model, including references to existing models, and the advantages of the study design. | Introduction |
| 3b. Specify the objectives, including whether the study describes the development or validation of the model. | 106-107 |
| METHODS |  |
| 4a. Describe eligibility criteria for participants and datasets. | 113-118 |
| 4b. Describe the origin of the data, and how the data were identified, requested, and collected. | 141-150 |
| 5. Explain how the sample size was arrived at. | 176-183 |
| 6a. Define the outcome that is predicted by the model, including how and when assessed. | 125-138+ 158-159 |
| 6b. Define all predictors used in developing or validating the model, including how and when measured. | Appendices A and B |
| 7a. Describe how the data were prepared for analysis, including any cleaning, harmonisation, linkage, and  quality checks. | 162 |
| 7b. Describe the method for assessing risk of bias and applicability in the individual clusters (eg, using  PROBAST). | NA |
| 7c. For validation, identify any differences in definition and measurement from the development data (eg,  setting, eligibility criteria, outcome, predictors). | 166-173 |
| 7d. Describe how missing data were handled. | 206-210 |
| 8a. Describe how predictors were handled in the analyses. | 196-203 |
| 8b. Specify the type of model, all model-building procedures (eg, any predictor selection and penalisation),  and method for validation. | NA |
| 8c. Describe how any heterogeneity across clusters (eg, studies or settings) in model parameter values was  handled. | NA |
| 8d. For validation, describe how the predictions were calculated. | 162-164 |
| 8e. Specify all measures used to assess model performance (eg, calibration, discrimination, and decision  curve analysis) and, if relevant, to compare multiple models. | 196-201 |
| 8f. Describe how any heterogeneity across clusters (eg, studies or settings) in model performance was  handled and quantified. | 201-203 |
| 8g. Describe any model updating (eg, recalibration) arising from the validation, either overall or for  particular populations or settings. | NA |
| 9. Describe any planned subgroup or sensitivity analysis, (eg, assessing performance according to sources of  bias, participant characteristics, setting). | 207-209 |
| RESULTS |  |
| 10a. Describe the number of clusters and participants from data identified through to data analysed. A flow chart may be helpful. | Table 1 |
| 10b. Report the characteristics overall and where applicable for each data source or setting, including the key dates, predictors, treatments received, sample size, number of outcome events, follow-up time, and amount of missing data. | Table 1 |
| 10c. For validation, show a comparison with the development data of the distribution of important variables (demographics, predictors, and outcome). | Table 2 |
| 11 Report the results of the risk of bias assessment in the individual clusters. | NA |
| 12a. Report the results of any across-cluster heterogeneity assessments that led to subsequent actions during  the model’s development (eg, inclusion or exclusion of particular predictors or clusters). | NA |
| 12b. Present the final prediction model (ie, all regression coefficients, and model intercept or baseline estimate  of the outcome at a given time point) and explain how to use it for predictions in new individuals. | NA |
| 13a. Report performance measures (with uncertainty intervals) for the prediction model, overall and for each cluster. | Results |
| 13b. Report results of any heterogeneity across clusters in model performance. | Appendices B1 and B2 |
| 14. Report the results from any model updating (including the updated model equation and subsequent  performance), overall and for each cluster. | NA |
| 15. Report results from any subgroup or sensitivity analysis. | 243-244 |
| DISCUSSION |  |
| 16a. Give an overall interpretation of the main results, including heterogeneity across clusters in model  performance, in the context of the objectives and previous studies. | 255-282 |
| 16b. For validation, discuss the results with reference to the model performance in the development data, and  in any previous validations. | 255-275 |
| 16c. Discuss the strengths of the study and any limitations (eg, missing or incomplete data, non-  representativeness, data harmonisation problems). | 331-336 |
| 17. Discuss the potential use of the model and implications for future research, with specific view to  generalisability and applicability of the model across different settings or (sub)populations. | 323-328 |
| Other information |  |
| 18. Provide information about the availability of supplementary resources (eg, study protocol, analysis code, datasets). | 358 |
| 19. Give the source of funding and the role of the funders for the present study. | 361 |

**Table C.** TRIPOD-Cluster Checklist

| **Disease** | **ICD-10 (Quan 2005) (31)** | **CCI (pts) (Quan 2011) (32)** |
| --- | --- | --- |
| **Chronic pulmonary disease** | I27.8-9, J40.x–J47.x, J60.x–J67.x, J68.4, J70.1, J70.3 | 1 |
| **Rheumatic disease** | M05.x, M06.x, M31.5, M32.x–M34.x, M35.1, M35.3, M36.0 | 1 |
| **DM with chronic complications** | E10.2–E10.5, E10.7, E11.2–E11.5,  E11.7, E12.2–E12.5, E12.7, E13.2–  E13.5, E13.7, E14.2–E14.5, E14.7 | 1 |
| **Renal disease** | I12.0, I13.1, I31.2, N03.2–N03.7, N05.2–N05.7, N18.x, N19.x, N25.0, Z49.0–Z49.2, Z94.0, Z99.2 | 1 |
| **Congestive heart failure** | I09.9, I11.0, I13.0, I13.2, I25.5, I42.0, I42.5–I42.9, I43.x, I50.x. P 290 excluded-children | 2 |
| **Dementia** | F00.x–F03.x, F05.1, G30.x, G31.1 | 2 |
| **Mild liverdisease** | B18.x, K70.0–K70.3, K70.9,  K71.3–K71.5, K71.7, K73.x, K74.x,  K76.0, K76.2–K76.4, K76.8, K76.9,  Z94.4 | 2 |
| **Hemiplegia, paraplegi, tetraplegi** | G04.1, G11.4, G80.0- G80.2, G81.x,  G82.x, G83.0–G83.4, G83.9 | 2 |
| **Any malignancy,**  **including lymphoma, leukemia, melanoma, except other malignant neoplasm of the skin** | C00.x-C26.x, C30.x-C34.x, C37.x-C41.x, C43.x, C45.x-C58.x, C60.x-C76.x, C81.x-C85.x, C88.x, C90.x-C97.x | 2 |
| **Moderate or severe liver disease** | I85.0, I85.9, I86.4, I98.2, K70.4,  K71.1, K72.1, K72.9, K76.5, K76.6,  K76.7 | 4 |
| **AIDS/HIV** | B20.x–B24.x | 4 |
| **Metastatic solid tumor** | C77.x–C80.x | 6 |

**Table D.** Charlson Co-morbidity Index (CCI) (31-33).

ICD = International Classification of Diseases. DM = Diabetes Mellitus. AIDS = Acquired Immunodeficiency Syndrome. HIV = Human Immunodeficiency Virus.

**Figure A1.** Expected widths of confidence intervals (CI) for AURCO curve based on sample size.

**Figure A2.** Expected widths of confidence intervals (CI) for calibration curve based on sample size.

**Figure B1.** PIHCA score nonparametric calibration plot, by hospital

CPC = Cerebral Performance Category

KUH = Karolinska University Hospital

SKH = Skaraborgs Hospital

CStG = S:t Görans Hospital

**Figure B2.** GO-FAR 2 nonparametric calibration plot, by hospital

CPC = Cerebral Performance Category

KUH = Karolinska University Hospital

SKH = Skaraborgs Hospital

CStG = S:t Görans Hospital
